# MyoD Over-Expression Rescues GST-bFGF Repressed Myogenesis

**DOI:** 10.3390/ijms25084308

**Published:** 2024-04-13

**Authors:** Shu-Hsin Fan, Ning Li, Kai-Fan Huang, Yun-Ting Chang, Chuan-Che Wu, Shen-Liang Chen

**Affiliations:** Department of Life Sciences, National Central University, Jhongli 32001, Taiwan; fanny.fan1994@gmail.com (S.-H.F.); kappa1362@gmail.com (N.L.); ncu.108801012@g.ncu.edu.tw (K.-F.H.); tarzan11140@gmail.com (Y.-T.C.); jaywu929@gmail.com (C.-C.W.)

**Keywords:** muscle, MyoD, bFGF, cell cycle, myogenesis, differentiation

## Abstract

During embryogenesis, basic fibroblast growth factor (bFGF) is released from neural tube and myotome to promote myogenic fate in the somite, and is routinely used for the culture of adult skeletal muscle (SKM) stem cells (MuSC, called satellite cells). However, the mechanism employed by bFGF to promote SKM lineage and MuSC proliferation has not been analyzed in detail. Furthermore, the question of if the post-translational modification (PTM) of bFGF is important to its stemness-promoting effect has not been answered. In this study, GST-bFGF was expressed and purified from *E.coli*, which lacks the PTM system in eukaryotes. We found that both GST-bFGF and commercially available bFGF activated the Akt–Erk pathway and had strong cell proliferation effect on C2C12 myoblasts and MuSC. GST-bFGF reversibly compromised the myogenesis of C2C12 myoblasts and MuSC, and it increased the expression of *Myf5*, *Pax3/7*, and *Cyclin D1* but strongly repressed that of *MyoD*, suggesting the maintenance of myogenic stemness amid repressed *MyoD* expression. The proliferation effect of GST-bFGF was conserved in C2C12 over-expressed with *MyoD* (C2C12-tTA-MyoD), implying its independence of the down-regulation of *MyoD*. In addition, the repressive effect of GST-bFGF on myogenic differentiation was almost totally rescued by the over-expression of *MyoD*. Together, these evidences suggest that (1) GST-bFGF and bFGF have similar effects on myogenic cell proliferation and differentiation, and (2) GST-bFGF can promote MuSC stemness and proliferation by differentially regulating *MRFs* and Pax3/7, (3) MyoD repression by GST-bFGF is reversible and independent of the proliferation effect, and (4) GST-bFGF can be a good substitute for bFGF in sustaining MuSC stemness and proliferation.

## 1. Introduction

Trunk skeletal muscle is derived from the embryonic tissues called somites that form on both sides of the neural tube in a rostral to caudal fashion. A mature somite will give rise to sclerotome and dermomyotome first, and later the dermomytome develops into dermatome and myotome that become the precursors of dermis and skeletal muscle, respectively [1]. Cells in the sclerotome will later differentiate into the lineage of bone and tendon. The myotome is formed by cells migrating from the epaxial and hypaxial lips of the dermomyotome. Another group of dermomyotome cells originating from the central part join myotome and they become the life-long stem cells in the skeletal muscle. Progenitors from hypaxial lip also migrate ventrally and laterally to develop into SKM of limbs and ventral body wall. The transcription factors Pax3 and Pax7 are expressed in myogenic stem cells in the somites and postnatal SKM, and are critical for their self-renewal and proliferation [1]. 

Several signals, such as Wnt and FGF, released from the tissues surrounding somite have been found to enhance/drive the development of myogenic cells [2]. These myogenic signals act in combination or sequentially to induce the expression of myogenic regulatory factors (MRFs), including *Myf5*, *Myo*D, Myogenin, and *Mrf4*, so the somitic cells can be confined inside the myogenic lineage [3,4,5,6,7]. Upon stimulation of differentiation signals, MRF actively transcribe the expression of downstream myogenic genes, such as *Myogenin*, *Mef2c*, and *MHC*, so the cells can fuse into multinucleated myotubes responsible for neural signals’ stimulated contraction. Some myogenic signals induce both determination and differentiation of myogenic lineage via transcriptional and/or post-transcriptional regulations.

Basic fibroblast growth factor (bFGF) is expressed in the neural tube, myotome, and limb buds during embryogenesis, where the protein is majorly found inside the cells [8]. After birth, high level of bFGF protein can be detected in the basal lamina containing endomysium of the mature skeletal muscle, and its level is highly induced in the SKM of dystrophic SKM of Mdx mice, implying its involvement in the postnatal regeneration [9]. It was found that inclusion of bFGF in the culture medium maintained the proliferation of isolated satellite cells (the adult myogenic stem cells) in vitro, so it has now become a standard component of satellite cells’ culture medium [7]. Interestingly, bFGF mRNA is translated in the cytosol and its protein is transported by an unconventional mechanism called “self-sustained translocation”, in which bFGF is phosphorylated by TEC kinase under the plasma membrane and induced to form oligomerized membrane pore by PI(4,5)P_2_, and finally attracted outside by heparan sulfate proteoglycans on cell surfaces [10,11]. This unique transportation process renders bFGF free of signal peptide; therefore, full length coding sequence (CDS) of bFGF can be expressed as a functional recombinant protein in bacteria. Besides, this unique transportation also endows bFGF with special post-translational modifications (PTM), distinct from proteins transported via the ER–Golgi endomembrane system [12]. 

Previous studies have identified the signalling mediators triggered by the binding of bFGF to its receptors FGFR1-4 on the cell surface. Receptor tyrosine kinases was activated by bFGF binding and subsequent activation of adaptor proteins, leading to activation of several signalling pathways, including PI3K/Akt and the mitogen-activated protein kinases (MAPK) pathways (reviewed in [13,14]). MAPKs activates various transcription factors, such as c-Jun and c-Fos, to enhance transcription of factors necessary for cell proliferation. Among them, Cyclin D1 seems to be the major driver mediating the bFGF cell proliferation effect [15]. Besides, a microRNA, miR29a-3P, was found to mediate part of the proliferation effect downstream of bFGF and is important for SKM regeneration after exercise and injury [16]. However, the transcription factors activated by bFGF signalling pathways to regulate the expression of *MRFs* and their target sites in *MRF* genes remain largely elusive.

It has been shown that expression of any MRF can induce the determination of myogenic lineage in stem cells and change the cell fates of other lineages into myogenic cells [17]. These findings point out the importance of the regulation of MRF expression by myogenic signals. Unfortunately, very few efforts have been contributed to discovering the regulation of MRF expression by myogenic signals, so current understanding of the mechanism by which myogenic signals activate MRF expression is very limited. Previously we have confirmed the induction of *MyoD* expression by Wnt3a via canonical and non-canonical pathways [18]. Canonical effector β-Catenin binds several conserved *cis*-elements in the core enhancer (CRE) region, while non-canonical effectors target *cis*-elements in other upstream regulatory regions (L fragment, −9481~−7972). It will be interesting to know what is the effect of bFGF on MRF and the MuSC factors Pax3 and Pax7, and if this effect on myogenic factors is coupled with that of cell proliferation. 

With the unique secretion pathway of bFGF, it is of interest to test if bacterially expressed GST-bFGF (fusion protein of glutathione S-transferase (GST) and bFGF) and commercially available bFGF have the same effect on the proliferation of myoblasts and MuSC. Furthermore, the proliferation effect of bFGF might be repressive to the expression and function of MRF, so it raises the concern of losing myogenic identity if myoblasts or MuSC are kept long-term under bFGF treatment. To answer these two questions, C2C12 myoblasts and MuSC were treated with GST-bFGF and bFGF, and similar effects of both proteins on cell proliferation and differentiation were found. Furthermore, we further confirmed that myogenic repression and cell proliferation are two independent effects of GST-bFGF, and myogenic identity can be conserved as GST-bFGF has a differential effect on various MRF. 

## 2. Results

### 2.1. Recombinant GST-FGF Induces Akt and MAPK Signalling in Myoblasts

**Figure 1:**
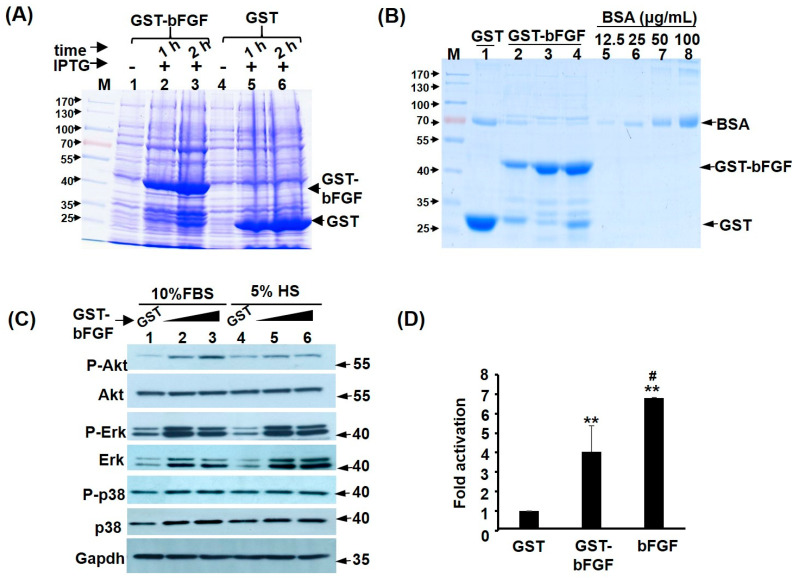
Recombinant GST-bFGF activates Akt–Erk pathways. (**A**,**B**) Recombinant GST and GST-bFGF proteins were induced and expressed in *E. coli* (**A**) and purified by glutathione beads, before being sterilized by passing through a filter with 0.45 μm pore size (**B**). Bovine serum albumin (BSA) was used to block non-specific adsorption of the filter and also as a protein level (12.5~100 μg/mL) reference. (**C**) C2C12 myoblasts in both growth medium (GM, 10% FCS) and differentiation medium (DM, 5% HS) were treated with GST (100 ng/mL) or GST-bFGF (50 and 100 ng/mL) for 24 h. The signaling proteins in total lysate were examined by Western blot, and the signal of Gapdh served as an input control. (**D**) The FGF reporter FIRE-luc was transfected into C2C12 and kept in GM with/without GST-bFGF and bFGF (50 ng/mL) for 48 h before being harvested for luciferase activity assay. The activity in GST treated cells was set as one-fold activation. **: *p* < 0.01 vs. GST. #: *p* < 0.05 vs. GST-bFGF. All experiments in this manuscript were repeated for >three times, unless otherwise indicated. The FIRE-luc treatment by bFGF was repeated twice, with triplicate in each assay.

The coding sequence (CDS) of mouse bFGF was cloned into the bacteria expression vector pGEX-4T1 to generate GST-bFGF fusion protein, which can be robustly induced by isopropyl-β-D-thiogalactoside (IPTG) (Figure 1A). After affinity purification with chromatography column and sterilization with filter, high purity GST-bFGF was obtained (Figure 1B, lanes 2~4: 3 independent purifications). The efficacy of this recombinant protein was tested by its ability to induce previously demonstrated cell proliferation signalling pathways, such as Akt and MAPK pathways, in C2C12 myoblasts (Figure 1C). We found highly increased phospho-Akt level by GST-bFGF in cells kept in growth medium (GM), but to a lesser level in cells kept in differentiation medium (DM). Phospho-ERK levels were highly increased by GST-bFGF, regardless of culture medium (Figure 1C). On the contrary, Phospho-JNK, -p38, and –mTOR levels were not significantly affected by GST-bFGF (Figure S1A). To confirm the effects of the activated signalling on the bFGF target genes, a previously reported FGF response reporter, FIRE-luc [19], was transfected into C2C12 myoblasts and we found high activity of this reporter in GM (Figure S1B), and it was still significantly activated by both GST-bFGF and bFGF treatments (Figure 1D). These results demonstrated the efficacy of recombinant GST-bFGF in triggering cell proliferation signalling pathways, and the Akt–ERK seems to be the major pathway mediating its effects inside the C2C12 myoblasts.

### 2.2. GST-bFGF Is a Strong Proliferation Inducer of Myoblasts

**Figure 2:**
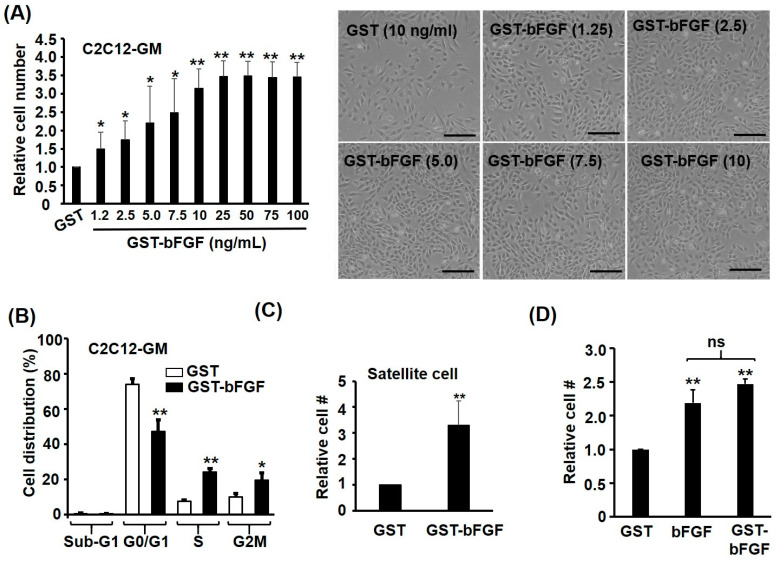
Recombinant GST-bFGF activates myoblast proliferation in GM. C2C12 myoblasts in GM were treated with GST or GST-bFGF protein for 48 h and their cell number (**A**) and cell cycle stages (**B**) were examined. Various doses (as indicated) of GST-bFGF were used in (**A**) and the morphology of cells (bright field under phase-contrast) after treatment (as indicated in ng/mL) is also shown in the right panel. Scale bar: 0.4 mm. (**C**) Primary satellite cells were treated as in (**A**), and the effect of GST-bFGF and bFGF was compared in (**D**). Cells in (**B**) were treated with 100 ng/mL GST-bFGF, but those in (**C**,**D**) were treated with 50 ng/mL GST-bFGF. Cell cycle stages were determined by flowcytometry after cells had been stained with propidium iodide (PI). * and **, respectively denoted *p* < 0.05 and *p* < 0.01 vs. GST treated cell. ns: non-significant. #: cell number.

In addition to activating proliferation signalling pathways, strong proliferative effect of GST-bFGF was also found in myoblasts kept in growth medium (GM) after 24–48 h (Figure 2A and Figure S1C). The minimal effective dose of GST-bFGF was found to be around 1.25 ng/mL, and a dose-dependent proliferation effect was observed from 1.25~25 ng/mL GST-bFGF on C2C12, and the effect became saturated in dosage higher than 25 ng/mL (Figure 2A). After being treated with GST-bFGF, there were more cells staying in S and G2M phases of the cell cycle, but less cells in the G0/G1 phase (Figure 2B), suggesting more cells were induced to go through cell division by GST-bFGF. It is of interest to know if the same proliferative effect can be seen in the adult muscle stem cells: accordingly, the satellite cells, and therefore primary satellite cells, were isolated, and strong proliferation of satellite cells induced by GST-bFGF was observed (Figure 1C). The proliferation effect of GST-FGF was further compared with that of commercially available FGF, and we found both proteins had a similar proliferation effect on C2C12 myoblasts (Figure 1D and Figure S1C). Taken together, these observations suggest that recombinant GST-bFGF can successfully induce cell proliferation signalling to trigger progression through cell cycle in myoblasts and satellite cells, which is similar to the effects observed with bFGF in previous studies and in our hands. Therefore, the recombinant GST-bFGF can be used as a substitutive growth factor for bFGF in stimulating myoblasts proliferation, and this protein is easy to be expressed and purified in a normal Molecular Biology laboratory.

### 2.3. GST-bFGF Reversibly Repressed Myogenic Differentiation

**Figure 3:**
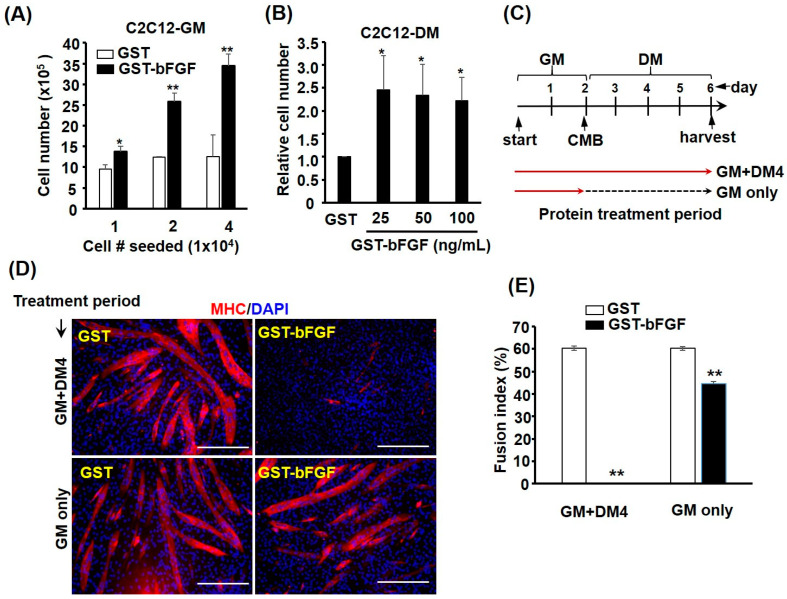
GST-bFGF reversibly represses myogenic differentiation. (**A**) The proliferation of C2C12 cells were seeded at various densities and their numbers after GST-bFGF treatment for 48 h were counted. (**B**) C2C12 myoblasts in DM were treated with GST or GST-bFGF protein for 48 h and their cell number were examined. (**C**) Schematics showing the GST-bFGF treatment. Myoblasts were treated with GST-bFGF at the proliferation stage, when kept in growth medium (GM only) or in both proliferation and differentiation stages (GM + DM4). Cells of both treatments were harvested on DM4 and were stained for MHC to examine their morphology (**D**) and fusion index (**E**). * and **: *p* < 0.05 and *p* < 0.01 vs. GST-treated cells. Scale bar: 0.25 mm.

Although bFGF has been routinely used as a growth factor for isolated satellite cells in vitro, its effect on myogenic differentiation has seldom been discussed. Myoblasts normally slow down/stop proliferation after they reach confluent, and the change of medium to differentiation medium (DM) will induce their differentiation. Interestingly, the proliferative effect of GST-bFGF was cell density-dependent, in which higher density showed stronger response (Figure 3A), implying community effect via autocrine positive feedback after GST-bFGF treatment, which also overcome the confluence-imposed growth inhibition. The growth inhibition was further found to be overcome by confluent cells kept in DM, suggesting the prevention of cell cycle exit of differentiating cells by GST-bFGF (Figure 3B). To confirm the effect on differentiation, myoblasts were treated either at the proliferating phase only (GM only) or at both proliferating and differentiating stages (GM+4 days in DM, Figure 3C), and we found dramatic repression effects on myogenesis if cells were treated at both stages (Figure 3D). However, removal of GST-bFGF at the differentiation stage allowed the rescue of myogenic differentiation, suggesting its reversible effect on repressing myogenic differentiation (Figure 3E).

### 2.4. GST-bFGF Induces Cell Cycle Genes but Selectively Represses Myogenic Genes

**Figure 4:**
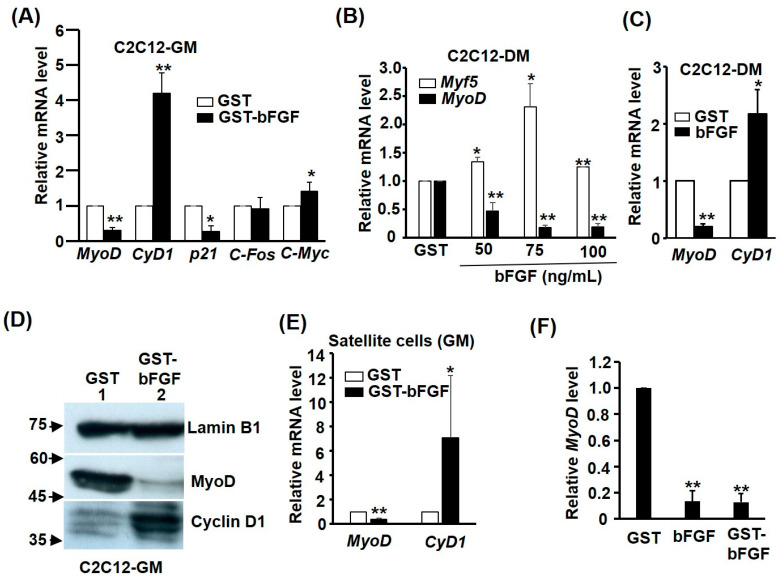
GST-bFGF signaling targets myogenic and cell cycle genes. The effect of GST-bFGF (50 ng/mL) on the expression of *MyoD* and cell cycle genes in C2C12 myoblasts kept in growth GM (**A**,**D**,**F**) or DM (**B**,**C**), and in satellite cells (**E**), and was examined by qRT-PCR. Myoblasts in (**B**) were treated with various doses of GST-bFGF in DM, as indicated. (**D**) The effect of GST-bFGF on *MyoD* and *Cyclin D1* protein levels in C2C12 cells kept in GM was examined with Western blot. The effect of GST–FGF and FGF on MyoD expression was compared in (**E**). The level of each gene in GST-treated cells was arbitrarily set as 1-old. * and **: *p* < 0.05 and *p* < 0.01 vs. GST treated cells.

The reversible effect on myogenesis prompted us to further investigate the regulation of myogenic and cell cycle genes by GST-bFGF signalling. In the GM, both the key myogenic genes *MyoD* and the CDK inhibitor *p21^cip^*^1^ were repressed, while the levels of cell cycle promoting genes *Cyclin D1* and *c-Myc* were significantly activated by GST-bFGF, demonstrating its myogenic repression but also the cell cycle-promoting effect via contrasted regulation of these two sets of genes (Figure 4A). The effects on *MyoD* and *Cyclin D1* were also similarly found in cells kept in DM, except for *Myf5*,which was surprisingly induced by GST-bFGF (Figure 4B,C). The contrasted regulation of *MyoD* and *CyclinD1* by GST-bFGF in cells kept in GM was also confirmed at the protein level (Figure 4D). The contrasted regulation of *MyoD* and *CyclinD1* by GST-bFGF was also confirmed in satellite cells (Figure 4E). It is also of interest to compare the effect of GST-bFGF and bFGF on *MyoD* repression, and similar effect of both proteins was observed (Figure 4F). These observations demonstrate that *MyoD* is consistently targeted by GST-bFGF, and also suggest the repression of *MyoD* expression by GST-bFGF might be the major cause of repressed myogenic differentiation (Figure 3D,E). However, it is unknown if *MyoD* repression is required for the confluence- and DM-resistant proliferation effect of GST-bFGF.

### 2.5. MyoD Repression Is Dispensable for GST-bFGF Induced Cell Proliferation

**Figure 5:**
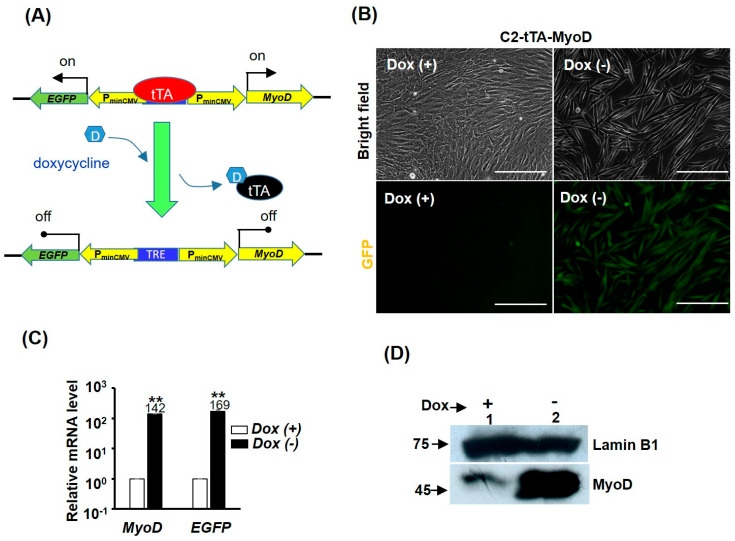
Establishment of *MyoD* over-expressed C2C12 stable clones. (**A**) Schematics showing the *TET-off* system, in which tTA binding to TRE is inhibited by doxycycline (Dox, 25 ng/mL). C2C12 stable clones (C2-tTA-MyoD) carrying *MyoD* over-expression system regulated by the *TET-off* system was established, and its morphology (**B**) and the expression of *MyoD* and *GFP* at mRNA (**C**) and protein (**D**) levels are shown. Scale bar: 0. 25 mm. **: *p* < 0.01 vs. Dox treated cells (Dox (+)).

**Figure 6:**
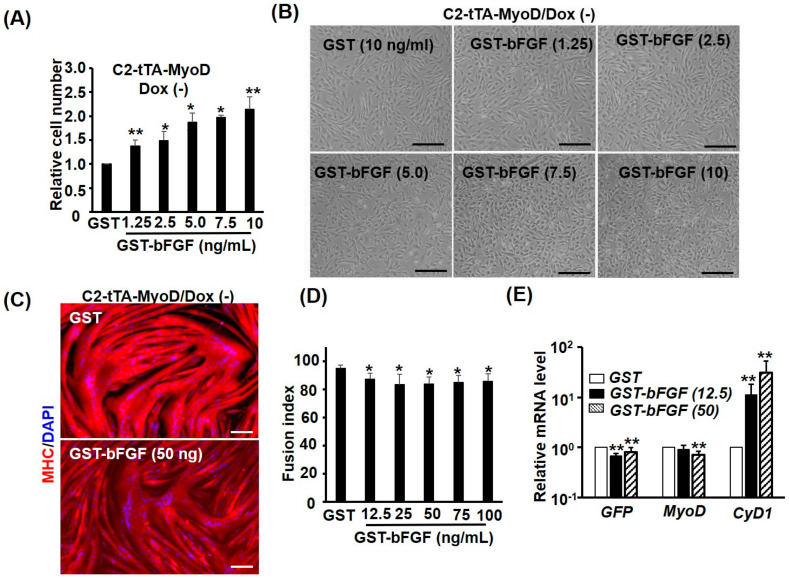
MyoD over-expression rescues GST-bFGF-repressed myogenesis. The effect of various doses of GST-bFGF on C2-tTA-MyoD proliferation when *MyoD* was over-expressed (Dox (−)) was examined (**A**), and their morphology is shown in (**B**). C2-tTA-MyoD myoblasts were treated with various doses of GST-bFGF during differentiation and their MHC-stained morphology (**C**), fusion index (**D**), and mRNA levels of *MyoD* and *Cyclin D1* (**E**) are shown here. Dox (+)/(−): with/without Doxycycline (25 ng/mL). * and **: *p* < 0.05 and *p* < 0.01 vs. GST treated cells. Scale bars in (**B**,**C**) are 0.4 mm and 0.2 mm, respectively.

As MyoD triggers myogenic differentiation and represses cell cycle at the same time, it is of interest to elucidate if repression of *MyoD* is necessary for GST-bFGF-induced cell proliferation. To answer this question, a *Tet-off* over-expression system was used to over-express *MyoD* in C2C12, in which *MyoD* and *EGFP* were highly activated by the transcriptional activator tTA, after doxycycline (Dox) was removed from the medium (Figure 5A–D). In cells over-expressed with *MyoD*, GST-bFGF-induced cell proliferation was conserved, implying that *MyoD* repression is independent of the cell proliferation effect (Figure 6A,B). We further found that *MyoD* over-expression induced strong myogenic differentiation, which was only marginally, although significantly, affected by GST-bFGF during this process (Figure 6C,D and Figure S2). The expression of *MyoD* in the *Tet-off* system was driven by the tTA-activated minimal *CMV* promoter constitutively; however, as both *EGFP* and *MyoD* level was marginally reduced by the GST-bFGF treatment during myogenesis after two days in DM (DM2), this suggests that their reduced level might be caused by the negative effect on the *Tet-off* system, either on the tTA activator or minimal *CMV* promoter. 

### 2.6. GST-bFGF Signaling Enhances the Stemness of Satellite Cells

**Figure 7:**
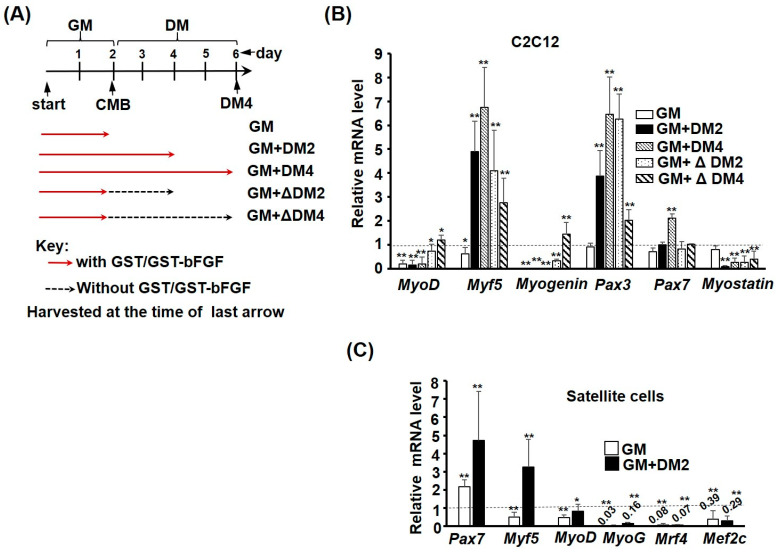
GST-bFGF signaling enhances the stemness of satellite cells. (**A**) Schematics showing the GST-bFGF treatment. C2C12 and primary satellite cells were treated with GST-bFGF at the proliferation stage and kept in growth medium (GM), or in both proliferation and differentiation stages (GM + DM2/DM4). Some cells treated in the GM were allowed to differentiate in DM for 2–4 days, without GST-bFGF (GM + ΔDM2/DM4). Cells were harvested after 2 days in GM or 2–4 days in DM, as indicated by the last arrow. (**B**,**C**) The expression of MRFs and Pax3/7 in C2C12 (**B**) and satellite cells (**C**) under different treatment patterns of GST-bFGF was detected with qRT-PCR, and compared with GST-treated control cells in pairs (relative to those in GST treated cells). The level of each gene in GST-treated cells was set as one-fold (not shown in the figure). * and **: *p* < 0.05 and *p* < 0.01 vs. GST treated cells.

As bFGF is widely used in the satellite cell culture and its repressive effect on myogenesis is reversible (Figure 3), it prompted us to examine the expression of myogenic genes during repressed myogenic differentiation by GST-bFGF, and also during the rescued differentiation after GST-bFGF removal. As expected, both *MyoD* and *Myogenin* expression was repressed by the presence of GST-bFGF, regardless of the stage or culture medium. However, their expression gradually recovered after removal of GST-bFGF (Figure 7A,B). Surprisingly, when cells were kept in DM, the expression of *Myf5* and *Pax3* was highly increased by GST-bFGF, and that of *Pax7* was also induced after 4 days in DM (DM4). Furthermore, after removal of GST-bFGF, the expression of *Myf5* and *Pax3* was still significantly induced after cells treated in GM were kept in DM without GST-bFGF for 2–4 days (GM + ΔDM2/4), indicating that signalling pathways triggered by GST-bFGF can be sustained in cells for a certain period after its removal, to induce the expression of stem cell factors. 

Myostatin is a myogenic repressor, and its expression was significantly repressed by GST-bFGF in all conditions when cells were kept in DM. The expression profile of these myogenic genes suggests that repression of *MyoD* and *Myogenin* is the main cause of myogenic repression by GST-bFGF; furthermore, other myogenic genes, such as *Myf5* and *Pax3*, were activated when myogenesis was initiated by serum withdrawal. The sustained repression of *Myostatin*, even after GST-bFGF removal, indicates the beneficial effects of GST-bFGF on myogenesis if it is only applied at the proliferative stage of myoblast.

Similar repression of *Mef2c* and most MRFs, including *MyoD*, *MyoG*, and *Mrf4*, was also observed in primary satellite cells kept in either GM or DM (Figure 7C). On the contrary, *Pax7* expression was highly induced by GST-bFGF, regardless of the culture condition. *Myf5* expression was only mildly activated in GM but consistently activated in DM, demonstrating that it is the major MRF-maintaining myogenic lineage in satellite cells cultured under GST-bFGF treatment. Together, these expression profiling in myoblasts and satellite cells suggests that GST-bFGF stimulates the stemness of satellite cells by activating myogenic stem cell markers *Pax3* and *Pax7,* while maintaining their lineage specification/determination by inducing the expression of *Myf5*, a MRF that is upstream to other MRFs.

## 3. Discussion

### 3.1. GST-bFGF Conserves the Property of Native bFGF

The growth factor bFGF was initially isolated from the brain and pituitary, and was found to be a potent mitogen to fibroblasts, chondrocytes, adrenal tumors, and glial cells [20,21]. Its proliferative effects on bovine myoblasts was later confirmed at the range of 0.1~1 μg/mL, but no effect was found on chick myoblasts [20]. However, delayed differentiation was observed in bovine myoblasts in the first study, and confirmed in many follow-up studies [7,22,23]. The repression of myogenic genes, such as *creatine kinase*, could be observed 22 h after the treatment of bFGF, indicating the involvement of transcription and translation of myogenic genes [22]. The proliferative effect of bFGF has been widely taken advantage of in the culture of isolated primary satellite cells, and is now a standard component of satellite cell culture medium, and also human embryonic stem cells [24,25].

In this study, the recombinant GST-bFGF protein was found to conserve the cell proliferative and myogenic repression activities, and this protein was therefore found to have very similar effect on myoblasts to the canonical bFGF. The fusion of GST and bFGF allows this fusion protein to be highly expressed in bacteria, and easily purified in large quantities. The conservation of cell proliferation and myogenic repression properties by GST-bFGF suggests that fusion with GST does not affect its binding with/recognition by receptor and other cofactors, such as heparan sulfate proteoglycans (HSPG), on the cells’ surface; therefore, GST-bFGF should be a reliable tool for studying the effects of bFGF on myogenesis of myoblasts and satellite cells. Furthermore, as the fusion of GST protein enhances the solubility and folding of bFGF in the *E. coli,* and serves as a convenient tag for affinity purification of the fusion protein, the production of GST-bFGF should be easier than that of bFGF for a general molecular biology laboratory. 

Most secreted proteins are transported to the cell surface via the canonical ER-Golgi-secretory vesicle pathway, and some of their amino acids are also modified during this outward transportation. Usually, these post-translational modifications (PTM) are important for their functions and/or stability after being released from the cells [26]. Interestingly, bFGF mRNA is translated in the cytosol and its protein is transported by the “self-sustained translocation” pathway, in which oligomerized bFGFs are translocated outside the cells and captured by heparan sulfate proteoglycans on cell surfaces [10,11], thus skipping off the ER–Golgi secretory vesicle pathway. Since most of the known PTM enzymes are located in the canonical pathway, it raises the question of if secreted bFGF contains any PTM. Recent studies have found multiple PTM in bFGF, including phosphorylation, acetylation, ubiquitination and sumoylation, but the effects of these PTM on the function of bFGF remains largely undefined [27,28,29]. Among the PTM, it was found tyrosines 82, 112, and 124 were phosphorylated by TEC kinase and the modification of tyrosine 82 was essential for its membrane translocation, as Y82A mutant failed to accumulate on the cell surface [12]. It was also found that rerouting bFGF to the classical secretory pathway results in PTM that blocks its binding to cell surface heparan sulfate proteoglycans, demonstrating the coupling of PTM and secretion pathway is essential for its function [30]. 

Our work with GST-bFGF suggests that the receptor binding and signalling induction are both conserved to induce cell proliferation and stemness, implying that PTM might not be required for these two events, if not all, and, even then, is only required for its secretion. However, this notion is contrary to the observation that S117A mutation drastically reduced its mitogenic activity, but without affecting its differentiation properties [31]. The same study also reported the nuclear translocation of wildtype and S117A bFGF into nucleus, and the uncoupling of its cell proliferation and differentiation activities will therefore provide interesting insight that will help to clarify if the same observation can be applied to GST-bFGF, when S117A mutation is included. Although GST-bFGF is already quite good at inducing cell proliferation and stemness, its efficacy can probably be further enhanced. It was found that the secretion and stability of bFGF could be enhanced by the mutations Cys70 and Cys88 to Ser and Asp, respectively, suggesting that efficacy of GST-bFGF might be further improved if these two mutations can be included [32]. 

### 3.2. Differential Effects on Free and Myofiber-Associated Satellite Cells

In the satellite niche of postnatal SKM, bFGF can be produced, by myofibers, fibroblasts, and satellite cells themselves [33,34,35], to regulate their proliferation and stem cell property. It was interesting to find that bFGF induce different responses in free and myofiber-associated satellite cells. Most studies on free satellite cells and cultured myoblasts found induced proliferation but repressed myogenic differentiation [7,22,23]. However, proliferation of satellite cells and their recruitment to associated myofibers were found to be induced by bFGF in primary myofiber culture [36], and the effect was also found to be enhanced in *Mdx* mice-derived myofibers [37]. The differential effects on free and myofiber-associated satellite cells suggest that factors in the micro-environment of satellite cells residence, i.e., the space between basal lamina and sarcolemma, might modulate satellite cells’ response to bFGF. For instance, we have found that GST-bFGF-repressed myogenesis can be rescued by the myogenic signal Wnt3a, demonstrating the combinatory effects of multiple signals might be very different from those of a single one.

The reduced *MyoD* level in C2C12 myoblasts and satellite cells treated with GST-bFGF argues that the satellite cells microenvironment (niche) in embryos might be different from that in the adult SKM (Figure 4). Actually, myogenic stem cells do not move to the satellite cell niche until embryonic day 16.5, which means that *MyoD* detected at this stage (12.5 dpc) is mainly expressed in myogenic cells located in the myotomes of developing somites [38]. It will be interesting to know if the *MyoD* expression in myogenic progenitors and developing myofibers respond differently to GST-bFGF while they are still in the myotome, where abundant bFGF is expressed. Furthermore, as satellite cells always associate with myofibers in vivo in postnatal SKM, and our results also demonstrate that GST-bFGF repressed myogenesis is reversible (which is probably due to activation of *Myf5* expression (Figure 3 and Figure 4) and reversible *MyoD* repression (Figure 7)), these evidences suggest that GST-bFGF should be a good agent for inducing satellite cell proliferation to promote/enhance SKM regeneration. In addition, the induction of *Myf5* in free myoblasts and satellite cells also suggests that myogenic lineage can be sustained after long-term proliferation in GST-bFGF treatment, while their myogenic stemness is also promoted by the induction of *Pax3/7* (Figure 7C).

### 3.3. MyoD Is the Major Target of bFGF in Myogenic Repression

Although the bFGF-induced delayed myogenic differentiation was identified several decades ago [20,39,40], the detailed mechanism was not analysed. The reversible myogenic repression and the strong repression of *MyoD* expression by GST-bFGF suggested that *MyoD* was the key target of GST-bFGF (Figure 4 and Figure 7). This hypothesis was confirmed when C2-tTA-MyoD was found to be resistant to the repressive effect and differentiate normally upon the challenge of GST-bFGF (Figure 6). However, the myotubes of C2-tTA-MyoD were still smaller and the fusion index was slightly lower than those treated with GST (Figure 6), implying that either other myogenic pathways are also targeted or MyoD functions at protein and mRNA levels are affected by GST-bFGF. The marginal reduction in *MyoD* mRNA level might provide some explanation of the observed smaller myotubes and lower fusion index, and the reduced MyoD might be caused by the reduced activity of the Tet-off system, either the tTA activator or minimal CMV promoter (Figure 6). Alternatively, the involvement of other pathways should be further considered, such as reduced *MyoD* mRNA stability or the trans-activational activity. Nevertheless, as the repressive effect of GST-bFGF on the myogenic differentiation and *MyoD* level of C2-tTA-*MyoD* cells is marginal, its effect on both mRNA stability or the trans-activational activity might also be marginal, suggesting that its repression of *MyoD* level might be majorly mediated by mechanisms reducing the transcription of the *MyoD* gene. Therefore, dissecting the effect of GST-bFGF on the *MyoD* gene promoter and enhancers is highly recommended in future study. Currently, the repression of *MyoD* promoter and enhancer activity by GST-bFGF has been confirmed, and we are dissecting the regulatory mechanism on targeted *cis*-elements. 

## 4. Materials and Methods

### 4.1. Plasmids

The coding sequence (CDS) of mouse *bFGF* gene was amplified from cDNA of embryonic mouse brain by *pfu* Taq DNA polymerase and inserted into the blunted *Sal*I site of the pGex-4T1 vector for expression of the GST-bFGF recombinant protein. The *Tet-off* expression vector pCEGFP-TRE-*MyoD* was created by inserting *Flag-MyoD* CDS into the blunted *Xho*I site of the pCEGFP-TRE vector [41]. The FGF reporter FIRE-luc was a generous gift from Dr. Ruey-Bing Yang (Institute of Biomedical Sciences, Academia Sinica). 

### 4.2. Cell Culture and Promoter Assay

Proliferating C3H10T1/2 fibroblasts (CCL-226, ATCC) and C2C12 myoblasts (CRL-1772, ATCC) were kept at low confluence in growth medium (GM, DMEM supplemented with 10% and 20%, respectively, fetal calf serum (FCS)). To induce myogenic differentiation, C2C12 at confluence myoblast stage (CMB) was changed to differentiation medium (DM, DMEM with 5% horse serum) for 4–6 days, until myotubes formed. For testing the effect of GST-FGF on C2C12 myoblasts in GM, the content of FCS was reduced to 10%, to avoid the possible interference from the growth factors in FCS. The FIRE-luc reporter was transiently transfected into C2C12 myoblasts in 12-well plates and kept in GM with GST or GST-bFGF for 24–48 h, being before harvested for luciferase assay using the Bio-Tek Clarity 2 luminometer, and the protein concentration of each well was then determined, for normalizing the results of luciferase assay. All transfection reactions were done in triplicate and repeated at least 3 times. The establishment of *Tet-off* stable clones has been described before [42]. Briefly, pCEGFP-TRE-*MyoD* was transfected into C2-tTA cells over-expressed with *tTA* gene, and selected with G418 and puromycin to generate C2-tTA-MyoD stable cones. C2-tTA-MyoD cells were kept in GM-containing antibiotics G418 (400 ng/mL), puromycin (2.0 μg/mL), and doxycycline (25 ng/mL), and the *MyoD* expression was induced by removing doxycycline from the medium. 

### 4.3. Recombinant protein Expression and Purification

The expression vector pGex-4T1-bFGF was transformed into the Rosetta strain of *E. coli* bacteria and the expression of *GST-bFGF* was induced at 18–23 °C for 6–12 h by 0.1 mM IPTG. Bacteria pellet was resuspended in PBS (pH7.3)-containing protease inhibitors before being sonicated in several cycles to break the bacteria until the lysate became clear. Unsoluble components were removed by centrifugation, and the GST-bFGF protein in the supernatant was captured by adding glutathione beads (fast-flow, Pharmacia), and incubated overnight. Then, glutathione beads were washed 3 times in buffer C (10 mM HEPES, 100 mM KCl, 5 mM EDTA, 2 mM MgCl_2_, 2 mM DTT, 20% glycerol, pH 7.3), and the GST-bFGF protein was eluted with elution buffer (50 mM Tris-HCl, pH 7.0, 20 mM MgSO_4_, 10 µg/mL heparin, 10–50 mM glutathione). The GST-bFGF protein buffer was changed to DMEM by flowing through the PD-10 desalt column, and further sterilized by passing through a low protein-binding PES filter (0.45 μm). All buffers, except for DMEM, used in the purification contained protease inhibitors. 

### 4.4. Quantitative RT-PCR (qRT-PCR)

The detailed protocol of qRT-PCR has been described in our previous works [18,41]. Briefly, cells and embryos were solubilized in Solution D (4M guanidinium thiocyanate, 25mM sodium citrate, pH7.0, 0.5% sarcosyl, 0.1% β-mecaptoethanol), and then total RNA was extracted with series of phenol/chloroform mixture, according to the well-established protocol [43]. Their cDNA was synthesized by the Superscript III kit (Invitrogen, Carlsbad, CA, USA), according to the manufacturer’s protocol. The qPCR product was detected by SYBR Green reaction mix (RealQ Plus 2X Master Mix Green, AMPLIQON, Odense M, Denmark). All reactions were performed in a Rotor-Gene Q real-time PCR cycler (QIAGEN, Germantown, MD, USA), with an amplification program of 45 cycles. The qRT-PCR primer sequences used in this study are described in Supplementary Table S1. 

### 4.5. Western Blot

The detailed protocol of Western blot has been described in our previous papers [41,42]. Briefly, C2C12 cells were lysed in RIPA buffer-containing protease inhibitors. Total protein (50 μg) was resolved on SDS–PAGE gel, and then transferred onto PVDF membrane. After blocking with blocking buffer (PBS containing 0.5% Tween 20 and 5% milk), the membrane was hybridized by primary antibody at 4℃ overnight. HRP-conjugated secondary antibodies were added into the membrane and incubated at room temperature for 1 h. After being washed with PBS, the HRP signal was detected by an enhanced chemiluminescence kit and viewed with X-ray film. Here is the list of primary antibodies: MyoD (Clone MoAb 5.8A, BD Pharmingen, San Diego, CA, USA); Gapdh (GTX100118, GeneTex); Lamin B1 (ab16048, abcam); Phospho-JNK (sc-6254, Santa Cruz, Dallas, TX, USA); JNK (#3708, Cell Signaling, Danvers, MA, USA); P44/42-Erk1/2 (#9101, Cell Signaling); Phopho-Erk1/2 (#9102, Cell Signaling); Cyclin D1 (GTX108624, GeneTex); Akt (#4691, Cell Signaling); Phospho-Akt (Thr308; #9275, Cell Signaling); Phospho-Akt (Ser473; #4058, Cell Signaling); Phospho-mTOR (Ser2448; #5536, Cell Signaling); mTOR (#2972, Cell Signaling); Phospho-p38 (Thr180/Tyr182; #5348, Cell Signaling); p38 (#9212, Cell Signaling); Phospho-c-Fos (Ser32; #5348, Cell Signaling); c-Fos (#2250, Cell Signaling); c-Jun (#9165, Cell Signaling) Phospho-c-Jun (Ser63; #9261, Cell Signaling).

### 4.6. Satellite Cell Isolation and Culture

Satellite cells were isolated with the pre-plating protocol, as reported before [44], in our recent paper [45]. Briefly, neonatal FVB mice were euthanized with CO_2_ overdose and their SKM was isolated and cut into small pieces, before being digested with collagenase (2 mg/mL) and trypsin (0.5×) at 37 ^°^C for 2 h. Suspended cells and tissues were collected with centrifugation and further digested with new enzymes for 2 h at 37 ^°^C, until tissue clumps disappeared. Equal volume of plating medium (DMEM with 10% horse serum) was added and the mixture was filtered through cell strainers, to remove undigested tissues. Cells in the filtrate were collected by spinning and resuspended in growth medium (DMEM with 20% FCS and 3% chicken embryo extract), before being seeded onto dishes for attachment of adherent cells (named as pre-plating #1, PP1). After 1 h, suspended cells were transferred to new dishes (PP2) and incubated for 2 h, before being transferred to new dishes (PP3) for further incubation for 24 h. Then, cells were transferred to new dishes coated with collagen I/II (PP4) and incubated for 24 h, before being transferred again to collagen-coated dishes, to collected PP5 cells. Both PP4 and PP5 cells contained majorly satellite cells (>95%), and were further expended and used as satellite cells in various assays. The use of mice for isolating satellite cells and embryos was approved by the Institutional Animal Care and Use Committee of National Central University, with the approval numbers NCU-108-015 and NCU-109-012.

### 4.7. Flowcytometry

Cells were seeded at low confluence and allowed to grow for 2–3 days, before being harvested by trypsinization. After an extensive wash in cold PBS, cells were fixed in an increasing level of ethanol (final concentration: 70%), by adding 99% ethanol (150 μL) into cell solution (in cold PBS) for seven times, with vigorous pipetting being applied to avoid cell clumping. Fixation was allowed to proceed overnight, and cells were stained in propidium iodide (PI) staining solution (0.1% Triton X-100, 2.5 μg/mL PI, and 10 mg/mL RNAse A in PBS) for 30 min, before being filtered through a cell strainer (pore size: 35 μm), to remove cell clumps. The distribution of cells staying in various stages of cell cycle was analyzed in the Coulter Epics-XL Flow Cytometer (Beckman Coulter, Miami, FL, USA) with FL3 610 nm fluorescence, to detect DNA content in the cells. List Mode data in EXPO32 ADC Analysis software 1.0 was used to collect and analyze data. 

### 4.8. Statistical Analyses

All quantitative results were derived from experiments with at least three independent biological replicates, and the averaged mean of all replicates of each treatment was used as the representative value of that treatment, as shown in the respective figure. The difference between control and various treatments was examined with Student’s *t*-test. All tests were two-sided, and a probability value of <0.05 was considered statistically significant. For real-time PCR, each assay was performed in duplicate of two different inputs (1× and 2×), to ensure linear PCR amplification, with the mean of these two inputs being used as the value of that assay.

## 5. Conclusions

Using C2C12 myoblasts and primary MuSC, we have demonstrated that GST-bFGF and bFGF have similar effects on cell proliferation and differentiation, suggesting that PTM of bFGF might only play a minor role in its stem cell proliferation effect. We further found that GST-bFGF can promote MuSC stemness and proliferation by differentially regulating *MRFs* and Pax3/7, confirming that myogenic repression and cell proliferation are two independent effects of GST-bFGF. In addition, MyoD repression by GST-bFGF is found to be reversible and independent of the proliferation effect, and the differential regulation of MRF is seen to sustais the myogenic identity. When taken together, the conclusions of our study confirm that GST-bFGF can be a good substitute for bFGF, in sustaining MuSC stemness and proliferation.

## Data Availability

Data are contained within the article and Supplementary Materials.

## Supplementary Materials

The following supporting information can be downloaded at: https://www.mdpi.com/article/10.3390/ijms25084308/s1.
