# Supplementary material for: MyoD Over-Expression Rescues GST-bFGF Repressed Myogenesis
Source: Int J Mol Sci. 2024 Apr 13;25(8):4308. doi: 10.3390/ijms25084308 (PMC11050597; doi:10.3390/ijms25084308)
Supplement: Supplementary file 1 [file ijms-25-04308-s001.zip › ijms-2934500-supplementary.pdf]

Supplementary Figure S1

(A)

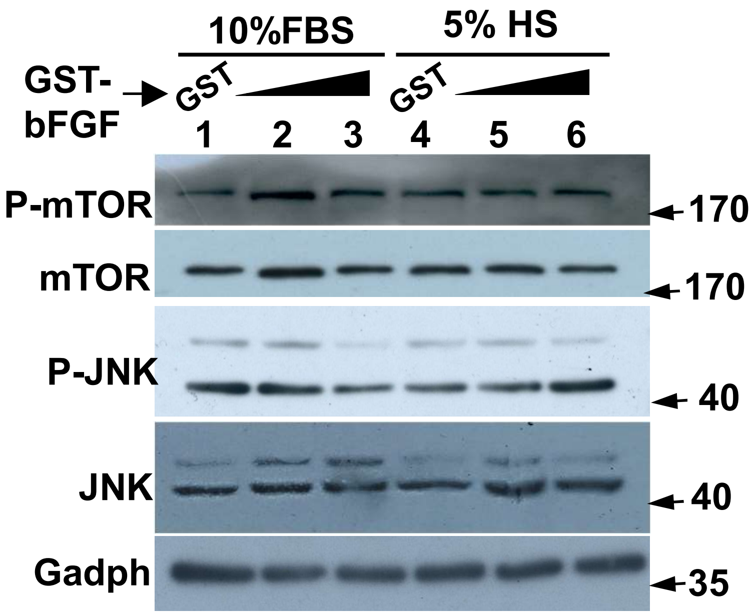

(C)

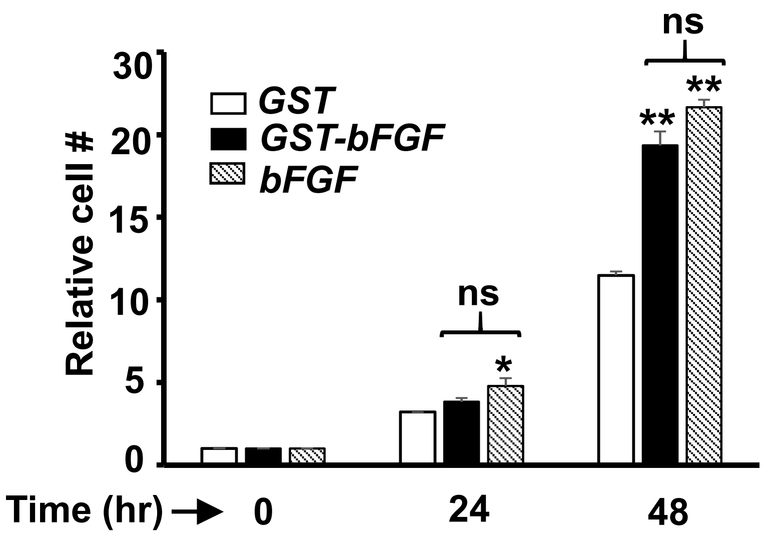

(B)

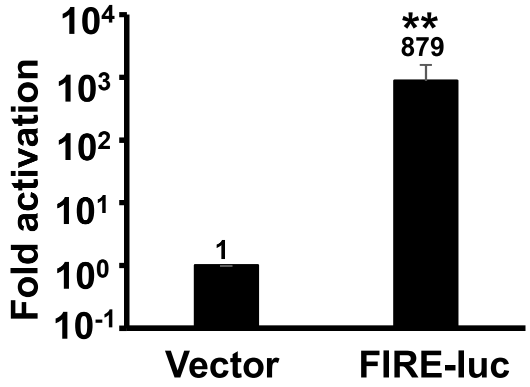

Supplementary Figure S1: The effect of GST-bFGF on cell signaling and proliferation. **(A)** C2C12 myoblasts in both growth medium (GM, 10% FCS)= and differentiation medium (DM, 5% HS) were treated with GST (100 ng/ml)= or=GST-bFGF=(50=and=100=ng/ml)=for=24=h.= The=signaling=proteins=in=total= lysate=were=examined=by=Western=blot=and=the=signal=of=Gapdh=served=as= input=control.=**(B)** The=pGL3-basic=vector=and=FGF=reporter=FIRE-luc=were= transfected into C2C12 and kept in GM with GST (50 ng/ml) for 48 hr before= harvested=for=luciferase=activity=assay.= The=activity=in=pGL3-basic=vector= transfected=cells=was=set=as=1-fold=activation.= **:=p<0.01=vs.=vector.= **(C)** C2C12=myoblasts=in=GM=were=treated=with=GST=or=GST-bFGF=protein=(25= ng/ml)=for=24~48=hr=and=their=relative=cell=numbers=at=24=and=48=hr=after= seeding=were=examined.=The=initial=cell=number=(time=0)=was=set=as=1-fold.=*= and=**:=p<0.05=and=p<0.01=vs.=GST=treated=cells.=N=2=for=this=proliferation= assay.

Supplementary Figure S2

C2-tTA-MyoD/Dox (-)

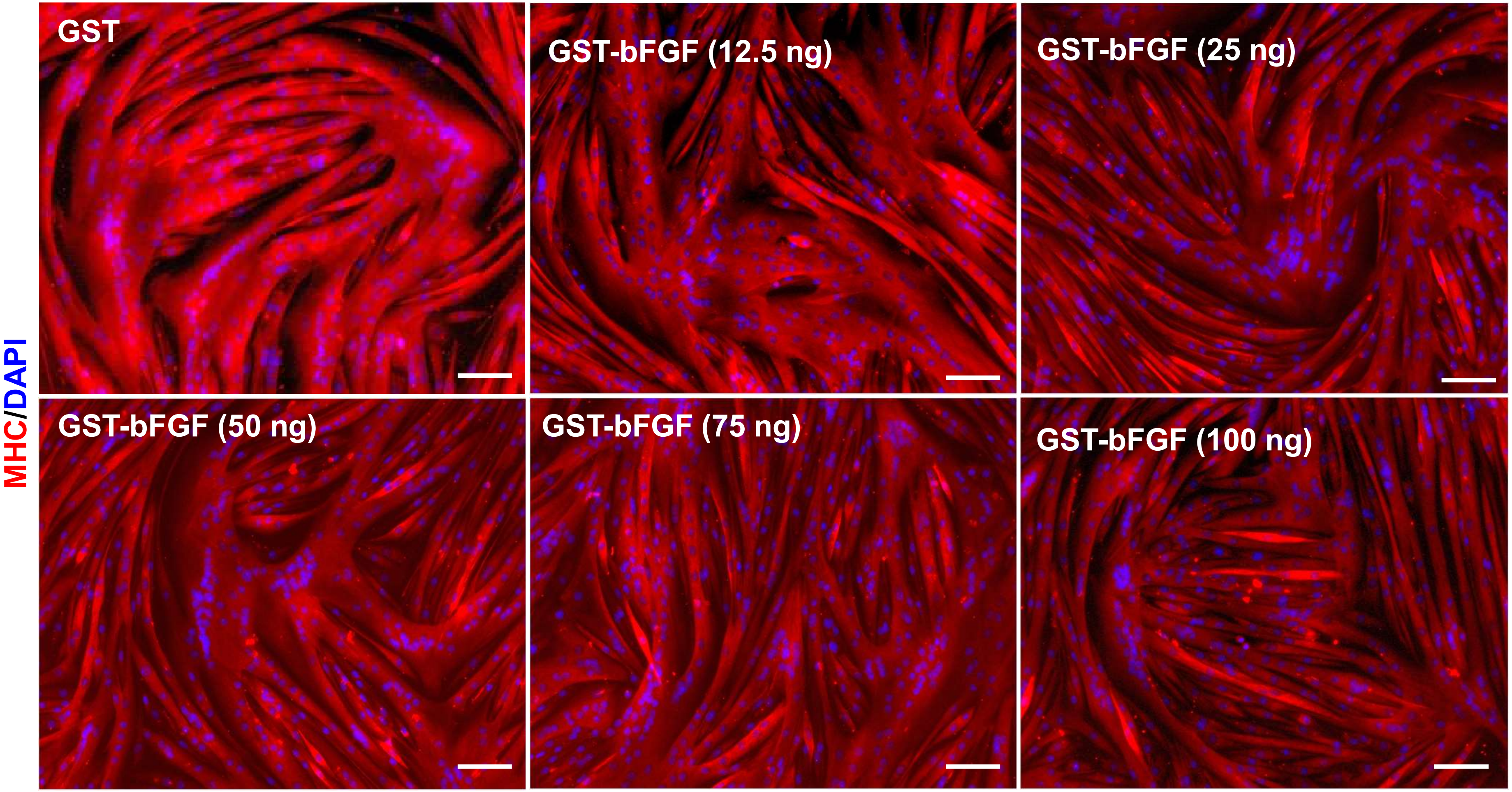

Supplementary Figure S2: The morphology of C2-tTA-MyoD myotubes.
C2-tTA-MyoD cells were induced to differentiation in DM and treated with various doses of GST-bFGF (as indicated) for 4 days in the absence of Dox.

Supplementary table SI: Primer sets used in the qRT-PCR and cloning.

**Gene name**	**Primer Sequence**	**NCU code**	**Amplicon Size (bp)**
m36B4	FP:5'-GAACGTGGGCTTCGTGTTCA-3'<br>RP:3'-AGCCTGGAAGAAGGAGGTCT-3'	014023<br>014024	167
MyoD	FP:5'-CTCCAACTGCTCTGATGGCATG-3'<br>RP:3'-CTCGACACAGCCGCACTCTTC-3'	002021<br>002022	135
Myf5	FP:5'-TGCACCACCACCAACCCTAA-3'<br>RP:5'-CAGGGCTGTTACATTCAGG-3'	015052<br>015053	196
Myogenin	FP:5'-CCAGTGAATGCAACTCCCACAGC-3'<br>RP:5'-AGACATATCCTCCACCGTGA-3'	006012<br>006013	166
c-Myc	FP:5'-GCTGGATTTCCTTTGGGCGT-3'<br>RP:5'-AATAGGGCTGTACGGAGTCG-3'	021018<br>021019	112
CyclinD1	FP:5'-TTGACTGCCGAGAAGTTGTG-3'<br>RP:5'-CTGGCATTTTGGAGAGGAAG-3'	054009<br>054010	154
p21	FP:5'-GCCGAAAACGGAGGCAGAC-3'<br>RP:5'-AAGATGGGGAAGAGGCCTCCTGA-3'	006007<br>006008	139
Pax3	FP:5'-ACTACCCAGACATTTACACCAGG-3'<br>RP:5'-AATGAGATGGTTGAAAGCCATCAG-3'	066021<br>066022	146
Pax7	FP:5'-TGGGGTCTTCATCAACGGTC-3'<br>RP:5'-ATCGGCACAGAATCTTGGAGA-3'	066007<br>066008	143
Myostatin	FP:5'-AGTGGATCTAAATGAGGGCAGT-3'<br>RP:5'-GTTTCCAGGCGCAGCTTAC-3'	066035<br>066036	144
c-Fos	FP:5'-CAGAGCGGGAATGGTGAAGA-3'<br>RP:5'-CTGTCTCCGCTTGGAGTGTA-3'	066001<br>066002	188
bFGF CDS (outer)	FP: 5'-ACACGGACTGGGAGGCTGGC-3'<br>RP: 5'-TGACAGTGTCTCAGTGACAGTGTCA-3'	0360010<br>0360011	615
bFGF CDS (inner)	FP: 5'-ATGGCTGCCAGCGGCATCACCTCGCTT-3'<br>RP: 5'-gtcgacGCTCTTAGCAGACATTGG-3'	036009<br>036006	468
